# Supplementary figures and images for: Additive genetic effect of GCKR, G6PC2, and SLC30A8 variants on fasting glucose levels and risk of type 2 diabetes
Source: PLoS One. 2022 Jun 3;17(6):e0269378. doi: 10.1371/journal.pone.0269378 (PMC9165855; doi:10.1371/journal.pone.0269378)

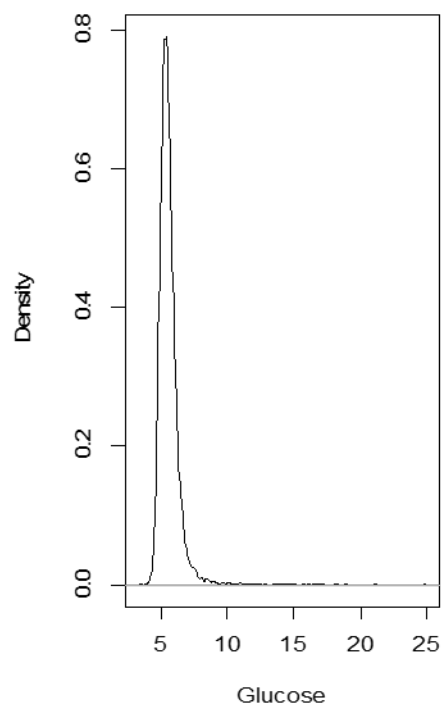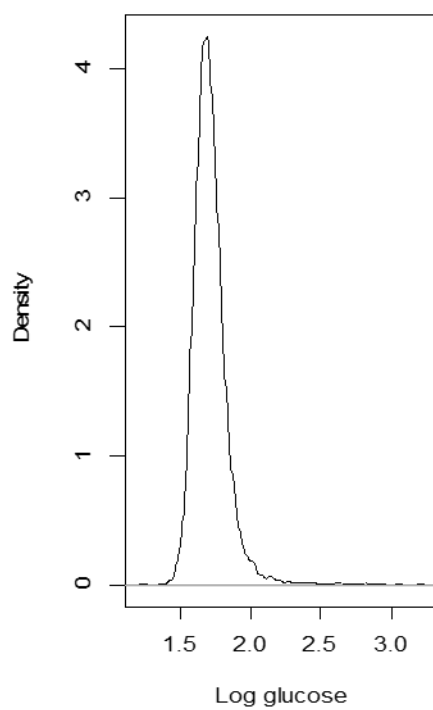

Supplement: S1 Fig — Untransformed (left) and log-transformed (right). (PDF) [file pone.0269378.s001.pdf]

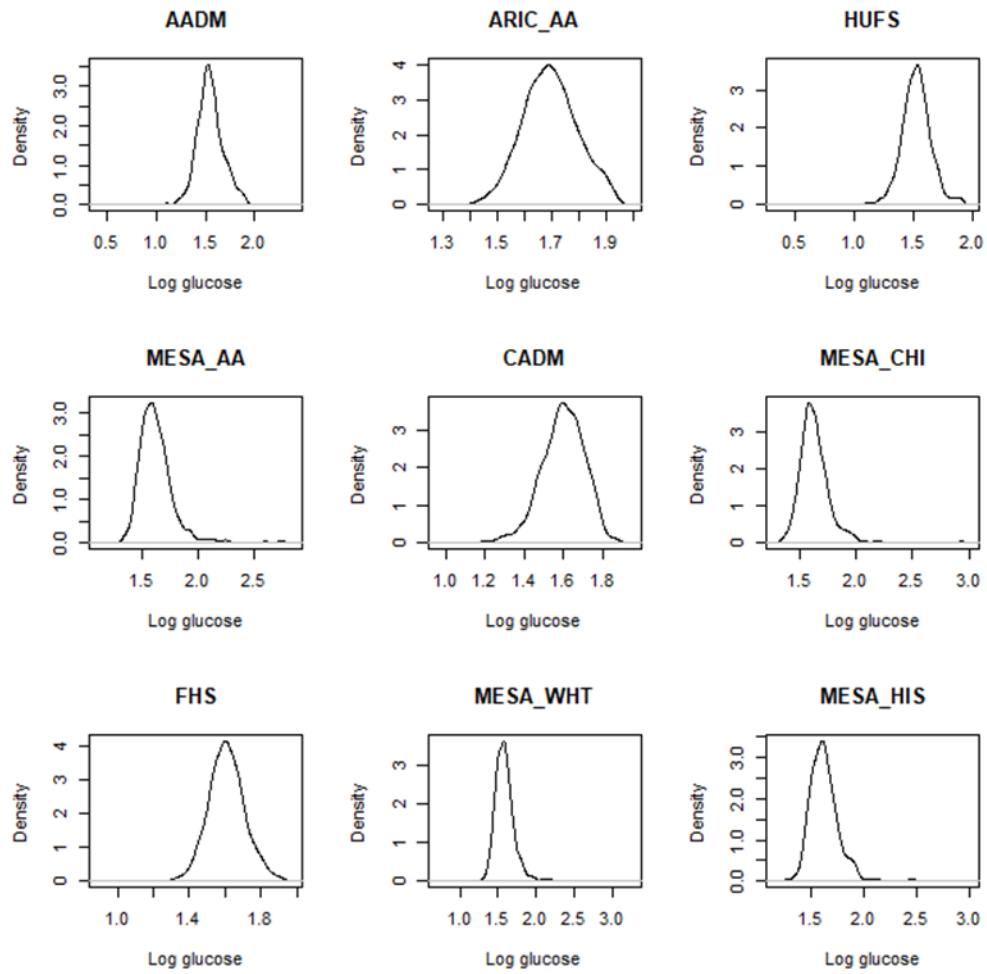

Supplement: S2 Fig — WHT, CHI, HIS, and AA refer to European Americans, Chinese, Hispanic Americans, and African Americans, respectively. (PDF) [file pone.0269378.s002.pdf]

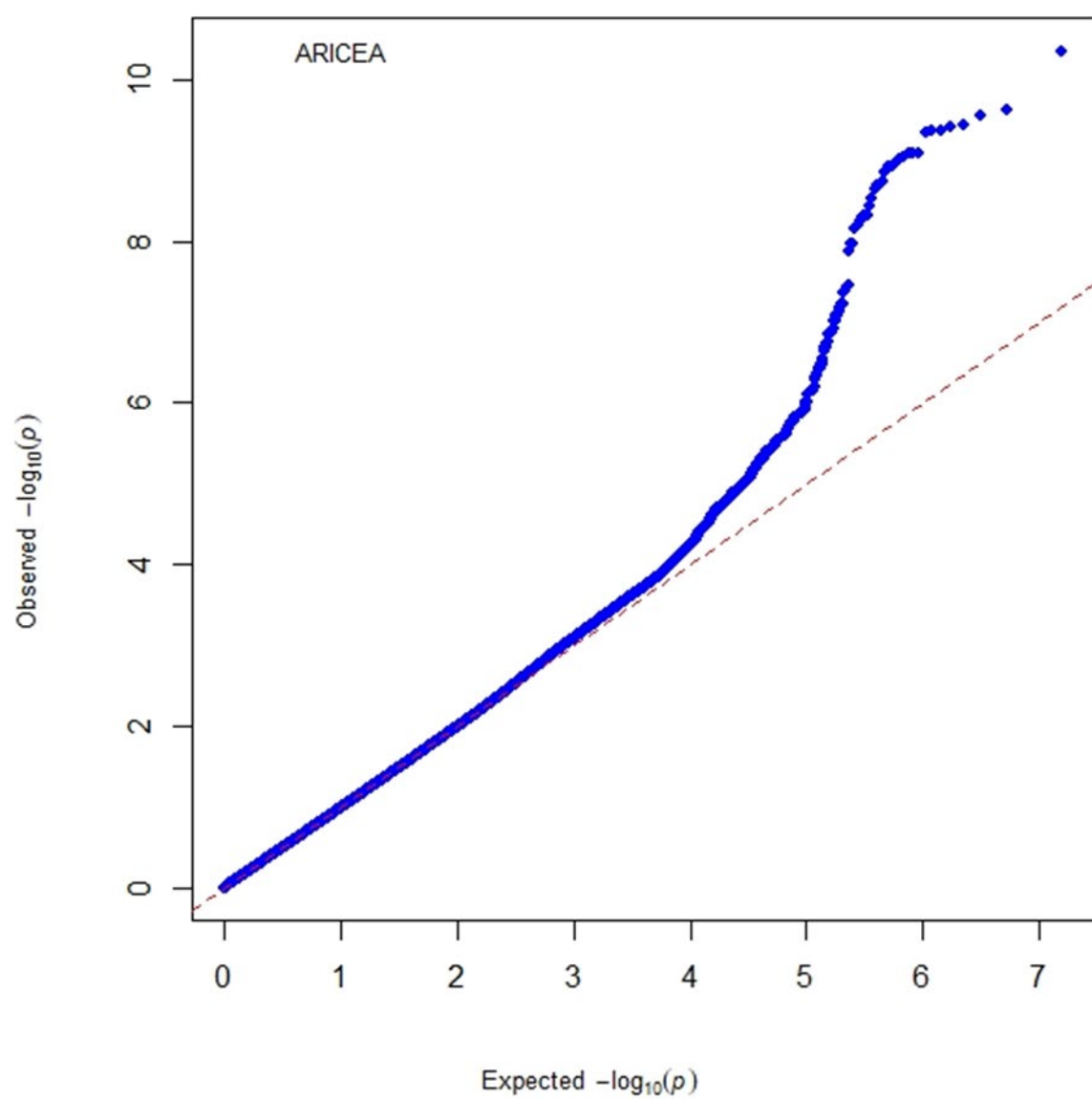

Supplement: S3 Fig — The x-axis represents expected p-values, and the y-axis represents observed p-values. All p-values are transformed as–log10(p-value). (PDF) [file pone.0269378.s003.pdf]

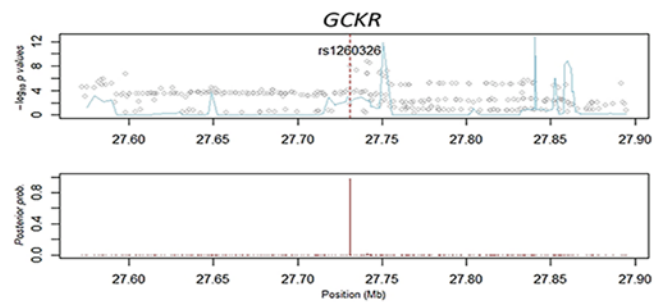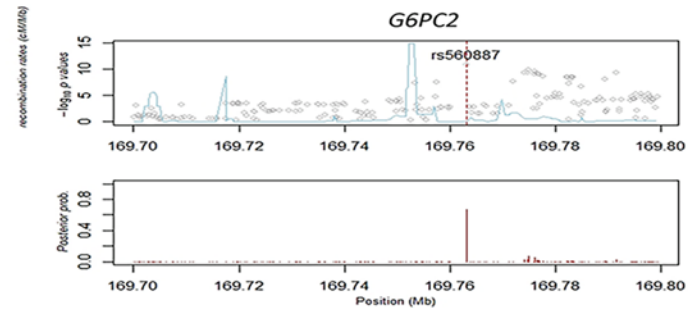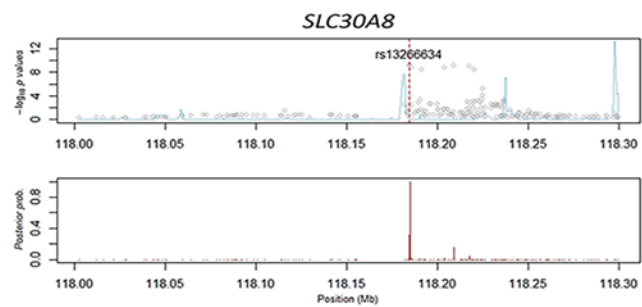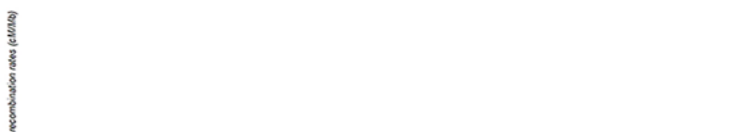

Supplement: S4 Fig — (Top) Region association plot: The x-axis represents position in Mb. The y-axis represents -log10 p-values. Sky-blue lines represent recombination rates (cM/Mb) from the 1000 Genomes Project. (Bottom) Posterior inclusion probabilities (PIP) based on fine mapping. The x-axis represents position in Mb. The y-axis represents PIP values. (PDF) [file pone.0269378.s004.pdf]

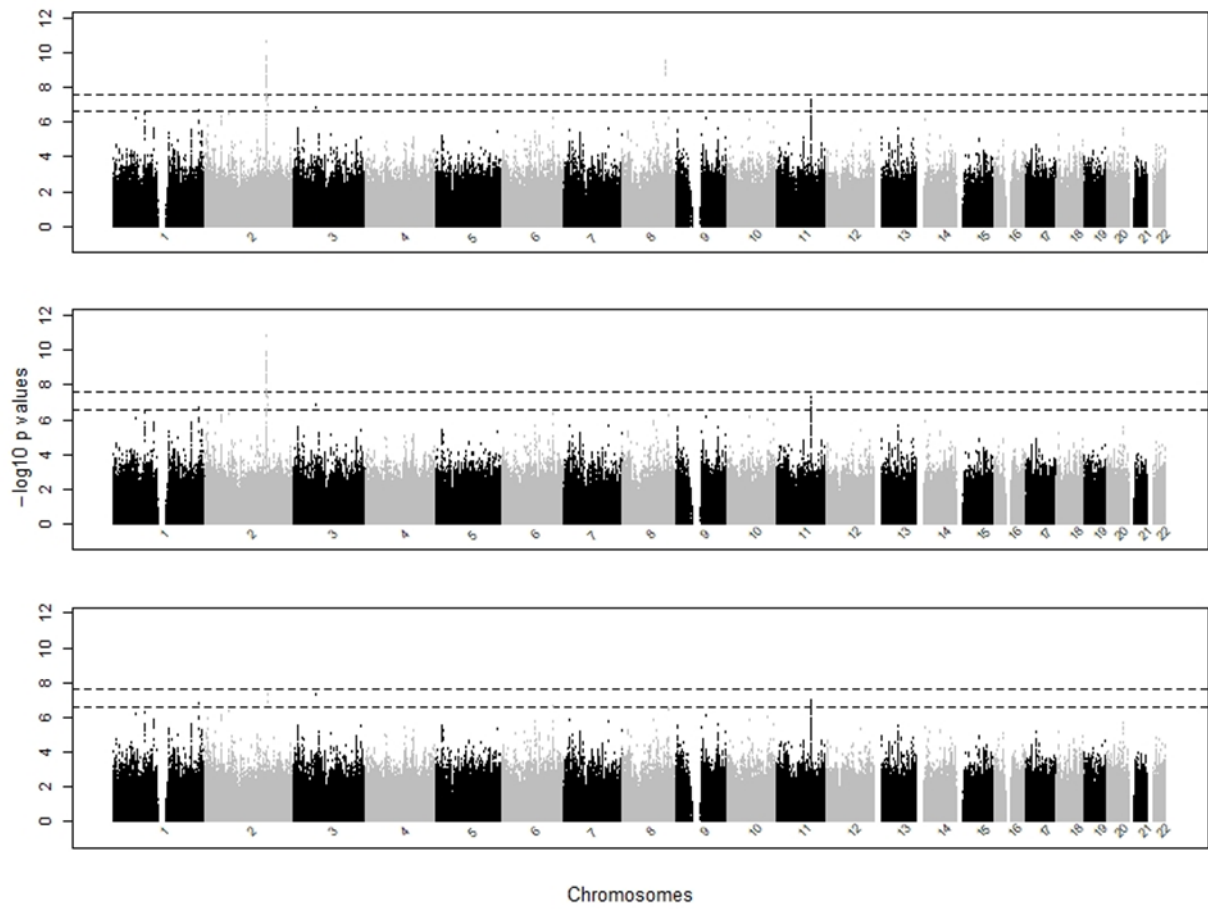

Supplement: S5 Fig — Row 1: Conditioning on rs1260326 (GCKR) abolished the peak at GCKR. Row2: Conditioning on rs1260326 (GCKR) and rs13266634 (SLC30A8) abolished the peaks at GCKR and SLC30A8. Row 3: Conditioning on rs1260326 (GCKR), rs560887 (G6PC2), and rs13266634 (SLC30A8) eliminated all genome-wide significant signals. (PDF) [file pone.0269378.s005.pdf]

# GCKR-L446P model 1

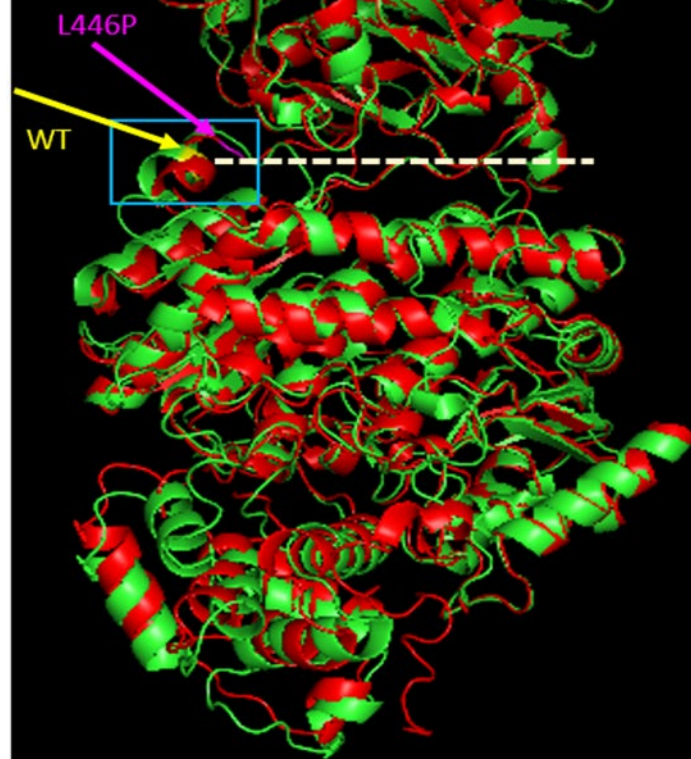

Supplement: S6 Fig — (PDF) [file pone.0269378.s006.pdf]

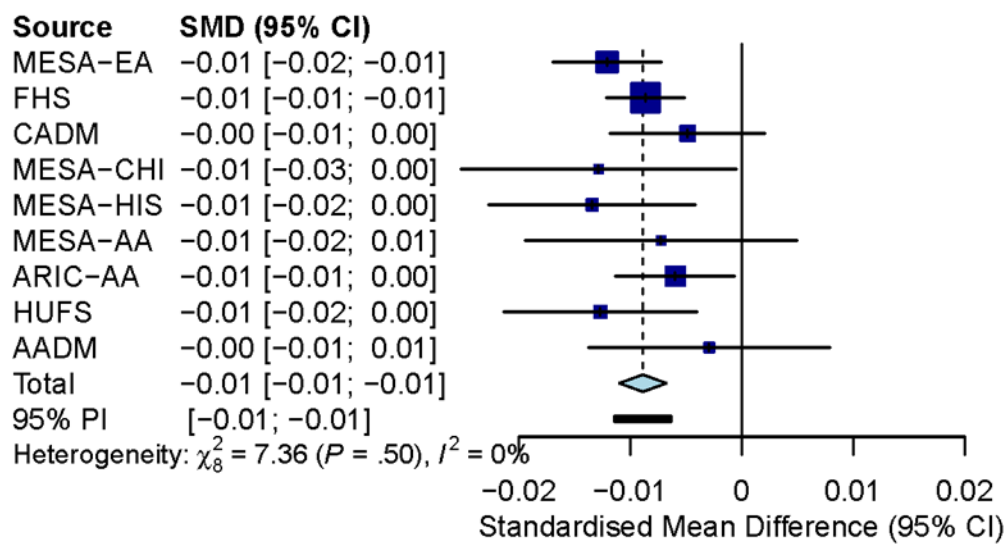

Supplement: S7 Fig — (PDF) [file pone.0269378.s007.pdf]

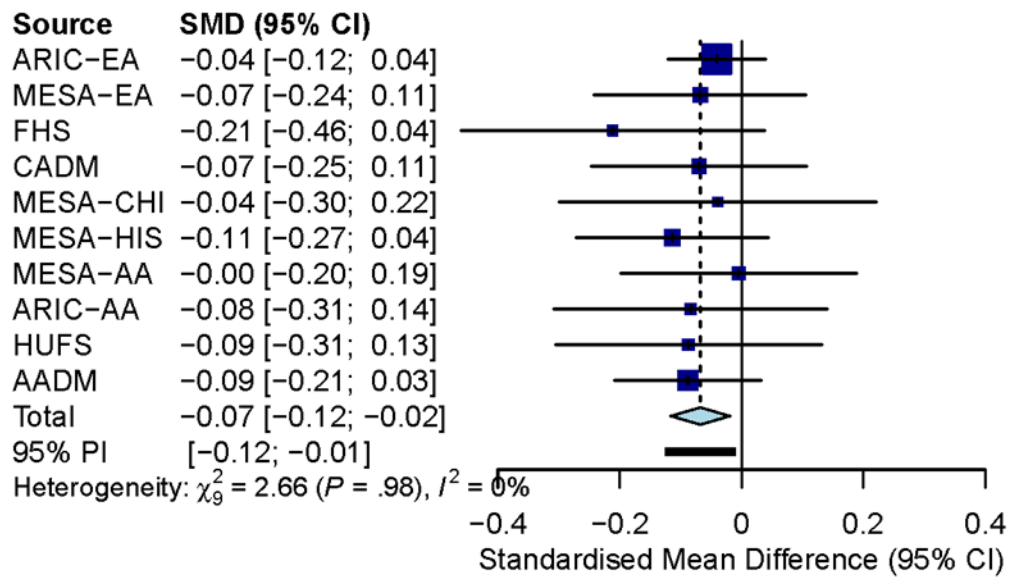

Supplement: S8 Fig — (PDF) [file pone.0269378.s008.pdf]
